# Three new species of ***Bolbochromus*** Boucomont (Coleoptera, Geotrupidae, Bolboceratinae) from Southeast Asia

**DOI:** 10.3897/zookeys.290.4696

**Published:** 2013-04-16

**Authors:** Chun-Lin Li, Ping-Shih Yang, Jan Krikken, Chuan-Chan Wang

**Affiliations:** 1 The Experimental Forest, National Taiwan University, Nantou 557, Taiwan, ROC; 2 Department of Entomology, National Taiwan University, Taipei City, Taiwan, ROC; 3 Naturalis Biodiversity Center, PO Box 9517, NL-2300 RA Leiden, Netherlands; 4 Department of Life Science, Fu Jen Catholic University, Hsinchuang, New Taipei City 24205, Taiwan, ROC

**Keywords:** *Bolbochromus*, new species, Geotrupidae, Bolboceratinae, Southeast Asia

## Abstract

Three new species of the Oriental bolboceratine genus *Bolbochromus* Boucomont 1909, *Bolbochromus minutus* Li and Krikken, **sp. n.** (Thailand), *Bolbochromus nomurai* Li and Krikken, **sp. n.** (Vietnam), and *Bolbochromus malayensis* Li and Krikken, **sp. n.** (Malaysia), are described from continental Southeast Asia with diagnoses, distributions, remarks and illustrations. The genus is discussed with emphasis on continental Southeast Asia. A key to species known from Indochina and Malay Penisula is presented. An annotated checklist of *Bolbochromus* species is presented.

## Introduction

The bolboceratine genus *Bolbochromus* Boucomont, 1909, is an Oriental genus that has a wide range and occurs eastward from Himalayan India and Sri Lanka to Southeast Asia, southern China, the Greater Sunda Islands, Philippines, Taiwan and its neighboring islands. A total of 19 species are currently known including three new species described here. Species of *Bolbochromus* inhabit forests, and the genus as here conceived is the most diverse bolboceratine group in Asia and it has never been systematically reviewed. For those species in Indochina, Paulian (1945) first diagnosed and recorded two species, *Bolbochromus laetus* (Westwood, 1852) and *Bolbochromus plagiatus* (Westwood, 1848), that were originally described from north India and Ceylon (presently Sri Lanka), respectively. We have examined a number of specimens looking like *Bolbochromus laetus* from Thailand and Vietnam. But Paulian’s record of *Bolbochromus plagiatus* in our view was based on misidentified specimens of the species described later (*Bolbochromus lao* Keith , 2012 from Laos and *Bolbochromus masumotoi* Ochi, Kon and Kawahara, 2011 from Cambodia), or to one of our new species described below. Paulian’s material was not traced and the type of *Bolbochromus laetus* is probably lost. Actually, specimens of *Bolbochromus* are not numerous in museum collections, probably due to inappropriate collecting methods. It is likely that the number of known *Bolbochromus* species will increase in the future when appropriate collecting methods are used.

Within the Bolboceratinae, adults of *Bolbochromus* are small (5.8–13.0 mm in length), glossy dorsally, pronotal midline indented, and body usually bicolored with brownish yellow or reddish brown markings on the surface of the pronotum and elytron which may inter/intraspecifically vary in number, size, and shape. The bicolored markings in *Bolbochromus* species, a character state that is rarely found in bolboceratine beetles, indicates a link with the genus *Bolbocerosoma* Schaeffer. However, the males of *Bolbochromus* lack tubercles on their pronotum as in the genus *Bolbocerosoma* (instead having an indented midline and/or transverse carina).

In this paper, we will improve the descriptions of generic characters based on Li et al. (2008), particularly those of the male genitalia (e.g., median lobe) which are of taxonomic and phylogenetic importance. Additionally, we provide an annotated checklist of the genus with the descriptions of three new species from Indochina and the Malay Peninsula, respectively.

## Materials and methods

All specimens used in this study were obtained on loan from the museums (names of curators are in acknowledgments) which are indicated in the type information of new species.

Specimens were studied and photographed using a Leica M205C stereo microscope with either a LED5000 MCI or HDI illuminator and a Canon 7D digital camera body. The measurements of specimens, preparation of aedeagus, and external morphological terms used in this paper follow Li et al. (2008). For those of the male genital structures, we employ the terms by D’Hotman and Scholtz (1990).

## Systematics

### Checklist of the genus *Bolbochromus* Boucomont

1. ***Bolbochromus catenatus* (Lansberge, 1886)**

*Bolboceras catenatum* Lansberge 1886: 135. Original combination.

**Distribution.** Sumatra (exact locality unknown); Borneo (exact locality unknown); Brunei (Boucomont 1914); Java (Boucomont 1914).

2. ***Bolbochromus celebensis* Boucomont, 1914**

*Bolbochromus celebensis* Boucomont 1914: 347. Original combination.

**Distribution.** Celebes (type locality: Toli-Toli).

3. ***Bolbochromus hirokawai* Ochi, Kon & Kawahara, 2010**

*Bolbochromus hirokawai* Ochi, Kon and Kawahara 2010: 97. Original combination.

**Distribution.** Negros Is. (type locality: Mt. Canla-on, Philippines).

4. ***Bolbochromus laetus* (Westwood, 1852)**

*Bolboceras laetus* Westwood 1852: 27. Original combination.

**Distribution.** Sri Lanka (exact locality unknown); Vietnam; Laos; S. China (Guizhou) (Paulian 1945, see our comment in introduction).

5. ***Bolbochromus lao* Keith, 2012**

*Bolbochromus lao* Keith 2012: 7. Original combination.

**Distribution.** Laos (type locality: Mt. Phou Sane, Khouang Province).

6. ***Bolbochromus lineatus* (Westwood, 1848)**

*Bolboceras lineatus* Westwood 1848: 356. Original combination.

**Distribution.** Sri Lanka (exact locality unknown).

7. ***Bolbochromus ludekingi* (Lansberge, 1886)**

*Bolboceras ludekingi* Lansberge 1886: 134. Original combination.

**Distribution.** Sumatra (exact locality unknown); Java (Boucomont 1914).

8. ***Bolbochromus malayensis* Li & Krikken sp. n.**

**Distribution.** Malay Peninsula (type locality: Ulu Gombak, Selangor State, Malaysia).

9. ***Bolbochromus masumotoi* Ochi, Kon & Kawahara, 2011**

*Bolbochromus masumotoi* Ochi, Kon & Kawahara 2011: 155. Original combination.

**Distribution.** Cambodia (type locality: Kbal Spean, Siem Reap).

10. ***Bolbochromus minutus* Li & Krikken sp. n.**

**Distribution.** Thailand (type locality: Khao Yai National Park, Nakhon Nayok Province).

11. ***Bolbochromus niger* Pouillaude, 1914**

*Bolbochromus niger* Pouillaude, 1914: 144. Original combination.

**Distribution.** Java (exact locality unknown).

12. ***Bolbochromus nigerrimus* (Westwood, 1852**)

*Bolboceras nigerrimus* Westwood 1852: 26. Original combination.

**Distribution.** N.India (Landour, Uttarakhand Province).

13. ***Bolbochromus nigriceps* (Wiedemann, 1823)**

*Scarabaeus nigriceps* Wiedemann 1823: 8. Original combination.

*Bolboceras sumatranus* Lansberge 1886: 135. Synonymized by Boucomont (1914: 347).

**Distribution.** Java (exact locality unknown); Sumatra (Boucomont 1914).

14. ***Bolbochromus nomurai* Li & Krikken sp. n.**

**Distribution.** N. Vietnam (type locality: Deo Pha Din, Son La Province).

15. ***Bolbochromus plagiatus* (Westwood, 1852)**

*Bolboceras plagiatus* Westwood 1852: 27. Original combination.

**Distribution.** N.India (type locality: Landour, Uttarakhand Province); Vietnam; Laos (Paulian 1945, see our comment in introduction).

16. ***Bolbochromus posticalis* (Westwood, 1852)**

*Bolboceras posticalis* Westwood 1852: 27. Original combination.

**Distribution.** Nothern (?) India (exact locality unknown).

17. ***Bolbochromus ryukyuensis* Masumoto, 1984**

*Bolbochromus ryukyuensis* Masumoto 1984: 168. Original combination.

**Distribution.** Taiwan; Iriomote and Ishigaki islands (type locality: Omotodake), S. W. Japan.

18. ***Bolbochromus sulcicollis* (Wiedemann, 1819)**

*Scarabaeus sulcicollis* Wiedemann 1819: 161. Original combination.

**Distribution.** Java (exact locality unknown).

19. ***Bolbochromus walshi* Pouillaude, 1914**

*Bolbochromus walshi* Pouillaude, 1914: 143. Original combination.

**Distribution**. Java (type locality: Soekaboemi and Toegoe).

#### Genus Bolbochromus

Boucomont, 1909

http://species-id.net/wiki/Bolbochromus

Bolbochromus Boucomont 1909: 117. Type species: *Bolboceras laetus* Westwood, 1852, by subsequent designation (Paulian 1945).

##### Description.

The following generic description is primarily based on the examination of continental *Bolbochromus* species. Length 5.8–13.0 mm. Dorsum glossy, color black to dark reddish brown, usually with brownish yellow or reddish brown markings on surface of pronotum and elytron varying in number, size and shape inter/intraspecifically. *Head*: Surface overall coarsely punctate. Clypeus with anterior border transversely arcuate or subtrapezoid with lateral border rounded; anterior margin unarmed or with tubercles/horns varying in number (1 or 3) and size. Frontal horn small to reduced. Antennal club with first antennomere mostly glabrous and polished on inner side; club ovoid in shape, apparently decreased in size apically, club segments slightly curved outwardly. Eye small in dorsal view, canthus broadened, rounded at anterior margin, entirely dividing eye into dorsal and ventral parts, ventral part larger than dorsal part. *Thorax*: Pronotum unarmed or with small anterior discal quadrituberculate carina; surface unevenly punctate, punctures usually large, deeply impressed at sides; form generally widest at middle, disc vaulted, apical declivity steep or gradually declined anteriorly; midline usually distinctly indented and punctate; lateral fovea poorly to moderately developed; anterior margin evenly arcuate; basal margin not beaded at middle. Middle coxae narrowly separated by metasternal process. *Elytron*: With 7 or 5 punctate striae between suture and humeral umbone, first stria curving along side of scutellum and reaching elytral base with first interval tapering basally; stria 5 not reaching base of elytron or vanishing together with stria 2 when intervals 2, 3 and 5, 6 fused completely; disc with 7, 5 or 3 impunctate intervals between suture and humeral umbone, longitudinally convex in varying degree, interval 2 usually more flat and narrower than others, interval 5 and 6 fused at base. *Legs*: Protibia with 6–10 contiguous teeth on outer margin. *Male genitalia*: Overall unevenly sclerotized, complex. Parameres symmetrically elongate or capsule-like in shape, membranous or well sclerotized laterally with median membranous parts, usually longer than basal piece, surface sparsely punctate, glabrous or setose with varying length of setae, apex usually rounded, in some species curved ventrally. Median lobe well developed, degree of sclerotization usually stronger than parameres, mostly trilobate and significantly varying in shape by species, trilobate median lobe consisting of dorsal sclerite and paired lateral sclerites articulated by paired supporting sclerites at base, lateral sclerites connected laterobasally to parameres. Internal sac embedded in median lobe, unarmed and hardly visible. Temones paired, tapered apically with articulation to base of median lobe, greatly varying in length, shape and degree of sclerotization interspecifically. Basal piece unevenly sclerotized, apical portion usually asymmetrical in shape. Genital capsule well developed.

##### Remarks.

*Bolbochromus* species shows little sexual dimorphism as compared with species of *Bolbelasmus* and *Bolbocerosoma*. The latter two genera have their major sexual dimorphisms in the frontal and pronotal protrusions. In contrast, the shape of frontal and pronotal protrusions in *Bolbochromus* species is identical between males and females. Both sexes in *Bolbochromus* species have slight morphological differences in the anterior margin of the labrum, the secondary punctures on the pronotal disc, and the apical tooth of the protibia, thus making it difficult to separate males and females.

##### Key to males of *Bolbochromus* species occurring in Indochina and the Malay Peninsula

**Table:** 

1	Body length larger than 7.9 mm	2
–	Body length smaller than 7.1 mm	4
2	Head with frontal horn; apical part of pronotal disc steep when viewed laterally	3
–	Head without frontal horn but with a weakly transverse convexity at base of vertex; disc of pronotum smoothly declined anteriorly when viewed laterally	*Bolbochromus lao* Keith
3	Frontal horn situated at middle between eyes, pronotum with yellowish triangle-shaped markings on each side, midline shallowly indented; elytral marking rounded in shape	*Bolbochromus masumotoi* Ochi, Kon & Kawahara
–	Frontal horn situated at middle between canthi; pronotum entirely brownish yellow with anterior slightly quadrituberculate carina, midline hardly indented or absent; elytra entirely brownish yellow or black, or disc surrounded by blackish markings	*Bolbochromus laetus* (Westwood)
4	Body length smaller than 5.8 mm; clypeal apex rounded; punctures of pronotal midline shallow and sparse	*Bolbochromus minutus* Li & Krikken, sp. n.
–	Body length larger than 6.8 mm; punctures of pronotal midline coarse and dense	5
5	Anterior margin of clypeus with a small, weakly-developed convexity at middle; vertex with an inconspicuous conical convexity at middle of base	*Bolbochromus nomurai* Li & Krikken, sp. n.
–	Anterior margin of clypeus completely beaded; vertex with an inconspicuous transverse carina at middle of base	*Bolbochromus malayensis* Li & Krikken, sp. n.

#### Bolbochromus minutus

Li & Krikken sp. n.

urn:lsid:zoobank.org:act:DBDAFF0E-EF40-4C98-91C6-D460D136F56C

http://species-id.net/wiki/Bolbochromus_minutus

**Figures 1–4.**
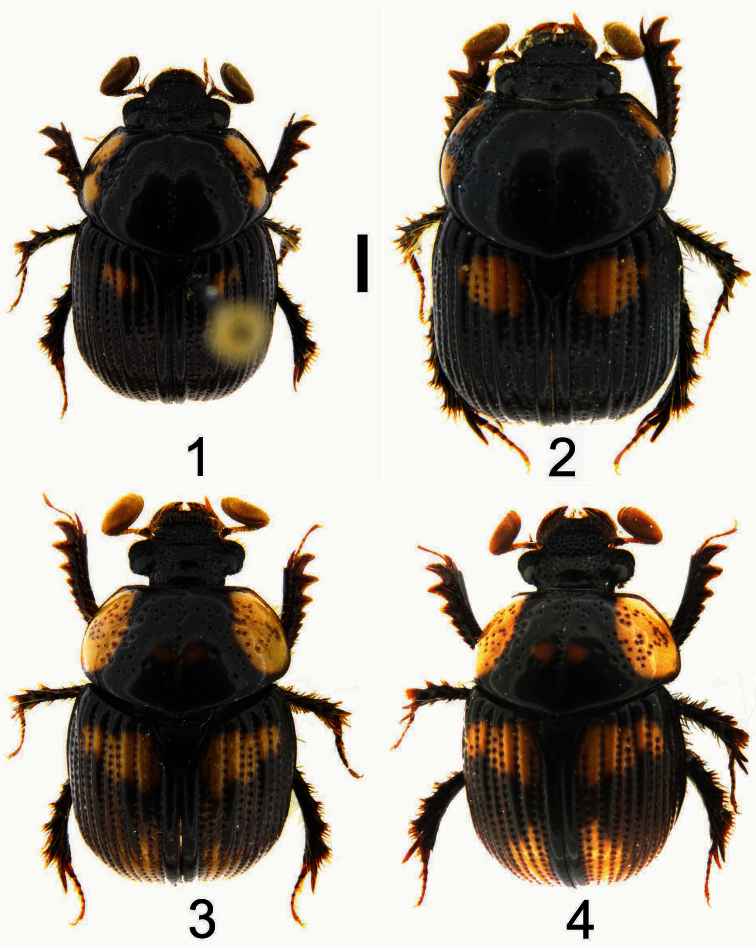
Dorsal habitus of *Bolbochromus* spp. **1** *Bolbochromus minutus* sp. n., holotype male **2** *Bolbochromus nomurai* sp. n., holotype male **3** *Bolbochromus malayensis* sp. n., holotype male **4** *Bolbochromus malayensis* sp. n., paratype female. Scale bar = 1.0 mm.

**Figures 5–12.**
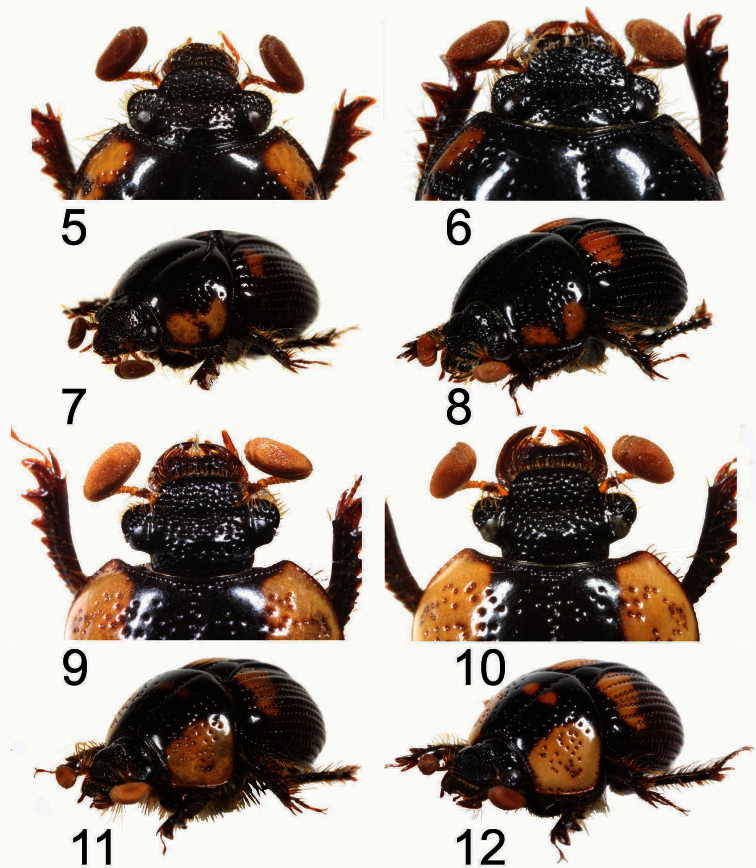
Dorsal view of head and left oblique view of *Bolbochromus* spp. **5, 7** *Bolbochromus minutus* sp. n., holotype male **6, 8** *Bolbochromus nomurai* sp. n., holotype male **9, 11** *Bolbochromus malayensis* sp. n., holotype male **10, 12** *Bolbochromus malayensis* sp. n., paratype female.

**Figures 13–18.**
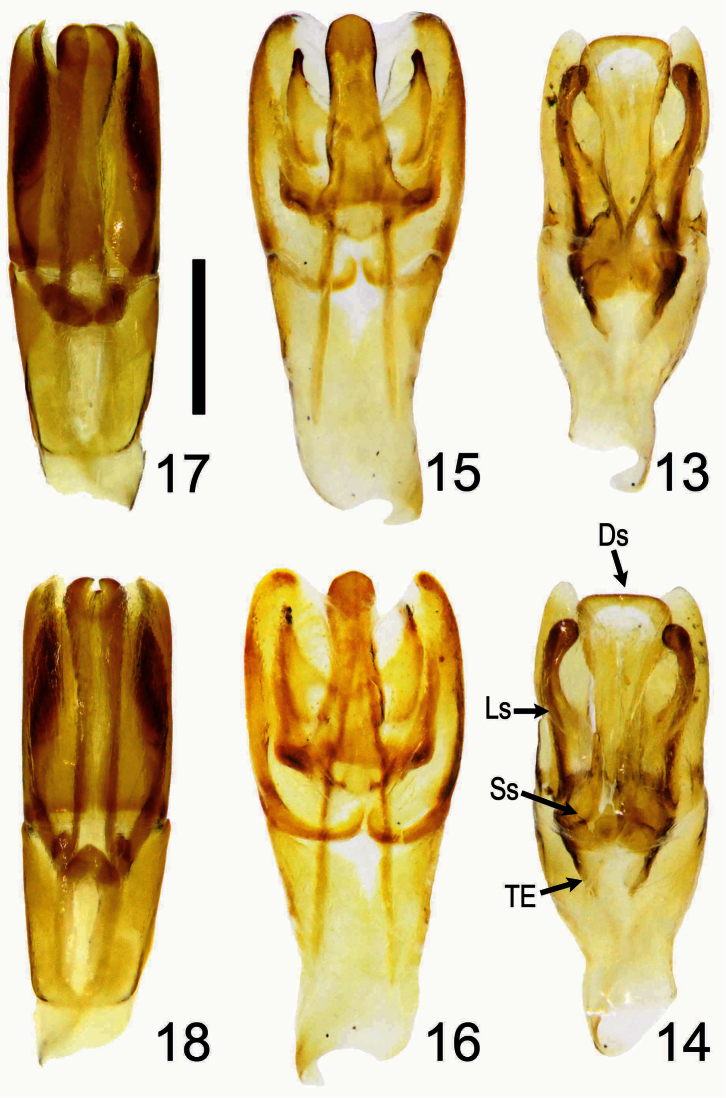
Male genitalia of *Bolbochromus* spp. **13–14** *Bolbochromus minutus* sp. n. **15–16** *Bolbochromus nomurai* sp. n. **17–18** *Bolbochromus malayensis* sp. n. **13, 15, 17** dorsal view; **14, 16, 18** ventral view. Ds, dorsal sclerite; Ls, lateral sclerite; Ss, supporting sclerite; TE, temones. Scale bar= 0.5 mm.

**Figures 19–22.**
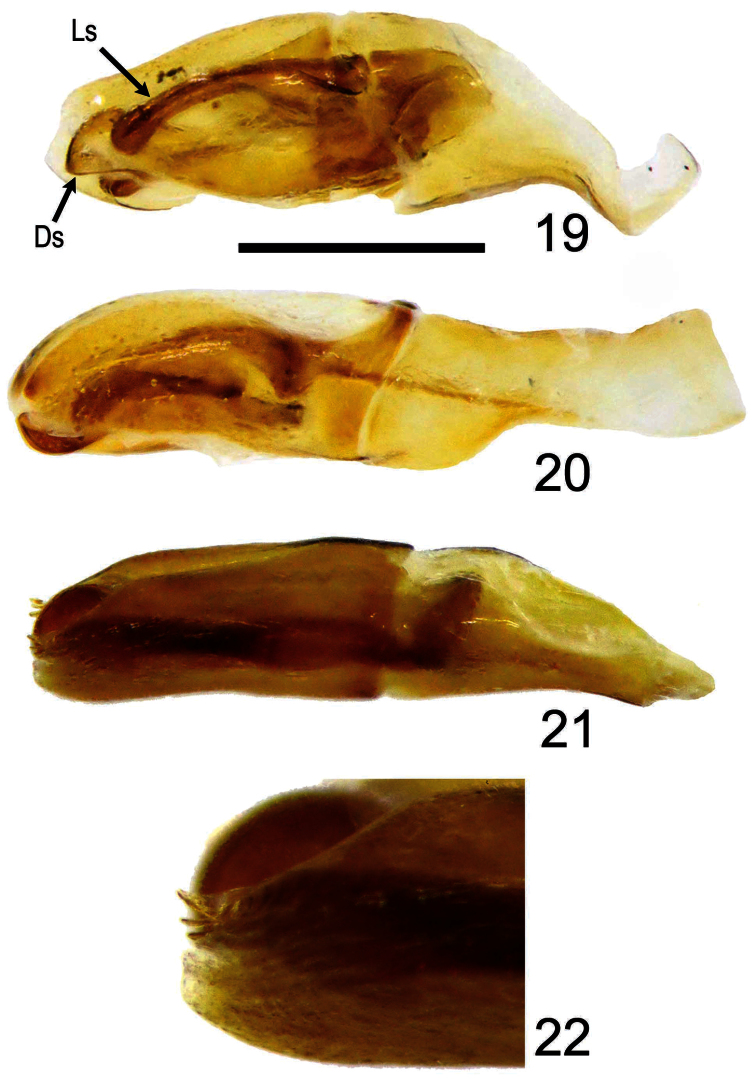
Lateral view of male genitalia of *Bolbochromus* spp. **19** *Bolbochromus minutus* sp. n. **20** *Bolbochromus nomurai* sp. n. **21** *Bolbochromus malayensis* sp. n. **22** ditto, tip of genitalia. Ds, dorsal sclerite; Ls, lateral sclerite. Scale bar = 0.5 mm for figures **19–21**.

Figs 1 , 5, 7 , 13–14 , 19

##### Holotype

male. THAILAND: Nakhon Nayok Prov.// Khao Yai Nat. Park., ca 700 m// 29. ix.–6. x. 1984// Karsholt, Lomholdt & Nielsen leg.//Zool. Mus., Copenhagen (deposited at the Universitetes Zoologiske Museum, Copenhagen, Denmark). The holotype (pinned) having both protarsi and right metatarsus broken.

##### Type locality.

**Figure 23.**
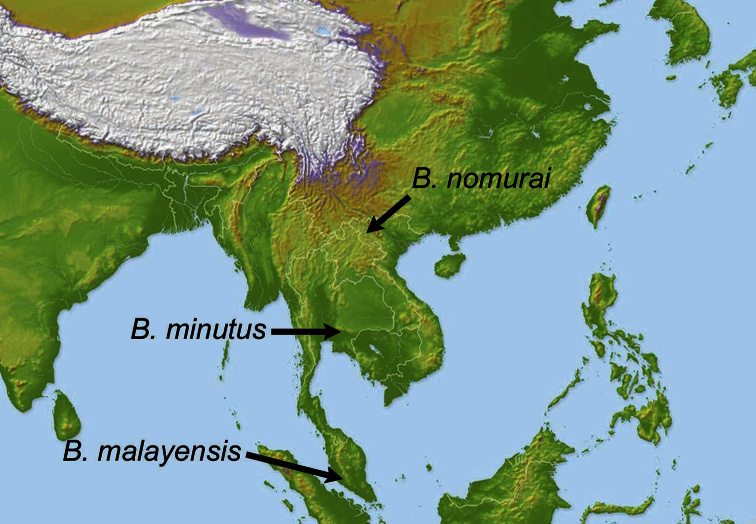
Distribution of the new *Bolbochromus* species.

Central Thailand: Nakhon Nayok Province, Khao Yai National Park, 14°26'N, 101°22'E (Fig. 23).

##### Description.

**Holotype Male** (Figs 1, 5, 7). Body length 5.8 mm; greatest width 3.5 mm. Form ovate, sides subparallel. Dorsum black with lateral margins of pronotum and elytron reddish black; irregular-shaped brownish orange markings located on sides of pronotum with exception of fovea (Fig. 7); size of elytral markings small, shape transversally irregular, across base of intervals 3–7, marking of interval 7 barely visible, (Fig. 1). *Head*:Labrum with anterior margin feebly triangularly concave centrally, sides notched. Clypeal apex rounded (Fig. 5), anterior margin beaded, surface coarsely punctate, punctures unevenly distributed, confluent or separated by less than 1 puncture diameter. Clypeofrontal suture absent. Vertex transversely, weakly convex at middle of base, punctures on surface more shallowly developed than those on clypeus, sparsely distributed. *Thorax*: Outline of pronotum generally rounded, surface coarsely punctate at center of lateral side of disc, with surrounding part impunctate, except for fovea; midline moderately indented, with shallow and inconspicuous punctures; both sides of midline and area in front of elytral base impunctate (Fig. 7); disc gradually declined anteriorly when viewed laterally (Fig. 7). Metasternal process poorly developed, narrowly separating middle coxae with anterior margin beaded. Scutellum with scattered secondary punctures, slightly longer than wide medially. *Elytron*:With 7 striae between suture and humeral umbone, stria 2 interrupted by stria 1 not reaching base, stria 5 terminating at basal one-ninth; interval 4 more convex and wider than others at basal one-fifth, interval 2, 5, and 6 less convex than others (Figs 1, 7). *Legs*: Protibia with 10 distinct teeth on outer margin, apical 3 teeth protruding, tip of apical tooth sharp and curved outwardly. *Male genitalia*: Length 1.6 mm. Parameres (Figs 13–14, 19) capsule-like, swollen overall when viewed laterally, weakly sclerotized laterally with medial and apical parts membranous; surface sparsely punctate, glabrous; longer in length than basal piece. Median lobe (Figs 13–14) trilobate; apex of dorsal sclerite largely swollen, shape rectangular; lateral sclerites downcurved (Fig. 19) with apex rounded swollen, more sclerotized and slightly shorter than dorsal sclerite; supporting sclerites elongate-oval. Internal sac invisible. Temones strongly sclerotized basally, shortly thickened to half of basal piece (Fig. 13). Basal piece with apical portion asymmetrical.

**Female**. Unknown.

##### Etymology.

The specific name is the Latin *minutus* which refers to the smallest body size of species currently known within *Bolbochromus*.

##### Diagnosis.

*Bolbochromus minutus* is similar to *Bolbochromus plagiatus*, but it can be distinguished based on the following combination of characteristics: smaller in body size (*Bolbochromus plagiatus* larger, body length approximately 6.3 mm); punctures of pronotal midline shallow and sparsely distributed (densely coarse rugopunctures in *Bolbochromus plagiatus*); elytral markings small across base of intervals 3-7 (large, across from stria 1 to epipleuron in *Bolbochromus plagiatus*); tip of protibial apical tooth sharp and elongate (obtuse and not elongate in *Bolbochromus plagiatus*).

##### Remarks.

Compared with other males in *Bolbochromus* species, *Bolbochromus minutus* can be easily separated from other similar species by the smaller body size, form of the elytral markings, and the punctures of the pronotal midline. In addition, the characteristics of the male genitalia are diagnostic.

#### Bolbochromus nomurai

Li & Krikken sp. n.

urn:lsid:zoobank.org:act:C0238EA6-41A8-4C65-BA14-BEAAA4DF697C

http://species-id.net/wiki/Bolbochromus_nomurai

Figs 2 , 6, 8 , 15–16 , 20

##### Holotype

male. The holotype is glued to a paper point and labeled: VIETNAM: Deo Pha Din (1000–1400m), Son La Prov.// [N. VIETNAM]// 24. VI. 1997// S. Nomura leg. (deposited at the National Museum of Nature and Science, Tokyo, Japan).

##### Type locality.

Northern Vietnam: Son La Province, Deo Pha Din, 21°70'N, 103°50'E (Fig. 23).

##### Description.

**Holotype male** (Fig. 2, 6, 8). Body length 7.1 mm; greatest width 4.1 mm. Form elongate-subovate, sides parallel. Dorsum black, with margins of head, pronotum, and elytron reddish black; isolated brownish orange markings located on each corner of pronotum, shape irregular, subequal in size (Fig. 8); elytral markings across base of striae 1-6 and interval 7, shape transversely rounded (Fig. 2). *Head*:Labrum with anterior margin feebly triangularly concave centrally, sides notched. Clypeal apex subtrapezoidal (Fig. 6), anterior margin beaded with a small, weakly-developed convexity at middle, surface smooth, coarsely punctate in uneven distribution, confluent or separated by less than 1 puncture diameter. Clypeofrontal suture absent. Vertex with inconspicuous conical convexity at middle of base with apex rounded, punctures on surface shallower and sparser than those on clypeus. *Thorax*: Outline of pronotum rounded, surface coarsely punctate along side of disc, less dense toward mid-disc; midline moderately indented with shallow and inconspicuous punctures; both sides of midline and area in front of elytral base impunctate with five smaller punctures at anterior end of midline (Fig. 6); disc gradually declined anteriorly when viewed laterally (Fig. 8). Metasternal process poorly developed, narrowly separating middle coxae with anterior margin beaded. Scutellum with scattered secondary punctures, slightly longer than wide medially. *Elytron*:With 7 striae between suture and humeral umbone, stria 2 interrupted by stria 1 not reaching base, stria 5 terminating at basal one-ninth; interval 4 more convex and wider than others at basal one-fifth, interval 2, 5, and 6 less convex than others (Figs 2, 8). *Legs*: Protibia with 10 distinct teeth on outer margin, apical 3 teeth protruding, tip of apical tooth downcurved. *Male genitalia*: Length 1.9 mm. Parameres (Figs 15–16) elongate, dorsal surface concave at basal half when viewed laterally, dorsal margin slightly declined at apical one-third anteriorly (Fig. 20), well sclerotized laterally with medial and apical parts membranous, surface sparsely punctate, glabrous; subequal in length to basal piece. Median lobe (Figs 15–16) trilobate; dorsal sclerite thumb-like with apex slightly swelling; lateral sclerites shorter than dorsal sclerite, broadly crescent-shaped, inwardly curved slightly with tip sharp and highly sclerotized; supporting sclerites L-shaped with central part more sclerotized than lateral side. Internal sac embedded in median lobe. Temones moderately sclerotized, thin and elongate to apical one-third of basal piece (Fig. 15). Basal piece with apical part asymmetrical.

**Female**. Unknown.

##### Etymology.

*Bolbochromus nomurai* is named after Dr. Shûhei Nomura of the National Museum of Nature and Science, Tokyo, who has always assisted C.-L. Li’s visits to the scarab collections of the museum.

##### Diagnosis.

*Bolbochromus nomurai* is similar to *Bolbochromus plagiatus*, but it can be distinguished based on the following combination of characteristics: larger in body size (smaller in *Bolbochromus plagiatus*); clypeal apex subtrapezoidal (rounded in *Bolbochromus plagiatus*); anterior margin of clypeus with a small, weakly-developed convexity at middle; (anterior margin simply beaded in *Bolbochromus plagiatus*); vertex with an inconspicuous conical convexity at middle of base (weakly convex in *Bolbochromus plagiatus*); outline of pronotum rounded (transverse in *Bolbochromus plagiatus*); punctures of pronotal midline shallow and inconspicuous (coarsely rugopunctate in *Bolbochromus plagiatus*); pronotal markings separated on each corner (connected in *Bolbochromus plagiatus*); elytral markings across base of striae 1–6 and interval 7 (across from stria 1 to epipleuron in *Bolbochromus plagiatus*).

##### Remarks.

Boucomont and Gillet (1921) and Paulian (1945) listed and diagnosed two *Bolbochromus* species (*Bolbochromus laetus* and *Bolbochromus plagiatus*) from Vietnam and neighboring areas which were originally recorded from Sri Lanka and northern India. Yet, we were not able to access the material studied by Paulian, and so we cannot be sure of the identity of specimens that he used. Considering the similarity of *Bolbochromus plagiatus* to *Bolbochromus nomurai* and other species occurring in Indochina, it is reasonable to assume that the identifications of Paulian are incorrect as it is unlikely he compared their male genitalia. To solve this problem it is necessary to re-examine the relevant specimens.

#### Bolbochromus malayensis

Li & Krikken sp. n.

urn:lsid:zoobank.org:act:BE6D04EE-CA30-4BF4-923C-E6260488D63E

http://species-id.net/wiki/Bolbochromus_malayensis

Figs 3–4 , 9–12 , 17–18 , 21–22

##### Holotype

male. The holotype is glued to a paper point and labeled: MALAYSIA: Selangor// Ulu Gombak// 21. V.–3. VI. 2003// Maruyama M. (FIT) (deposited at the National Museum of Nature and Science, Tokyo, Japan).

##### Paratype.

1 female, with the same collecting data as the holotype.

##### Type locality.

Western Malaysia: Selangor State, Ulu Gombak, 3°35'N, 101°78'E (Fig. 23).

##### Description.

**Holotype male** (Figs 3, 9, 11). Body length 6.8 mm; greatest width 3.8 mm. Form ovate, sides subparallel. Dorsum of head, pronotum, interval 1 (sutural interval), and base of elytron black with elytral striae and remaining intervals brownish black to yellowish brown; round, brownish yellow markings located on lateral third of pronotum, 2 additional minor marking at sides of basal one-third of midline (Fig. 11); elytral markings across base of striae 1–8 and interval 9, shape transversely irregular (Fig. 3). *Head*: Labrum with anterior margin feebly triangularly concave centrally, sides notched. Clypeal apex trapezoidal with lateral border rounded (Fig. 9), anterior margin beaded, surface rugosely punctate, confluent or separated by less than 1 puncture diameter. Clypeofrontal suture absent. Vertex with an inconspicuous convexity of carina at middle of base, coarse punctures on surface same as those on clypeus, moderately distributed. *Thorax*: Outline of pronotum transverse, surface coarsely punctate along side of disc, moderately dense; midline moderately indented with well-defined, coarse punctures; area in front of elytral base impunctate with coarse punctures at anterior one-third of sides of midline (Fig. 9); disc gradually declined anteriorly when viewed laterally (Fig. 11). Metasternal process poorly developed, narrowly separating middle coxae with anterior margin beaded. Scutellum slightly longer than wide medially, surface with 5 coarse punctures and scattered secondary punctures,. *Elytron*: With 7 striae between suture and humeral umbone, stria 2 interrupted by stria 1 not reaching base, stria 5 terminating at basal one-ninth; width of interval 3 and 4 same at basal one-fifth with interval 2, 5 and 6 less convex than others (Figs 3, 11). *Legs*: Protibia with 10 distinct teeth on outer margin, apical 3 teeth protruding, tip of apical tooth curved outwardly. *Male genitalia*: Length 1.7 mm. Parameres (Figs 17–18) elongate, dorsal margin slightly declined at basal one-fifth, becoming more declivous at apical one-fourth (Fig. 21), well sclerotized laterally with apical part membranous, surface almost impunctate, glabrous; subequal in length to basal piece. Median lobe (Figs 17–18) trilobate; dorsal sclerite vertically bilobed with apex notched; lateral sclerites elongate, equal in length to dorsal sclerite, overall highly sclerotized, apex tufted with 4 robust setae (Fig. 22); supporting sclerites kidney-shaped, evenly sclerotized. Internal sac embedded in median lobe. Temones membranous, thin and elongate to apex of basal piece (Fig. 17). Basal piece with apical portion asymmetrical.

**Paratype female** (Fig. 4, 10, 12). Similar to holotype male with minor differences of lighter body color, secondary punctures on pronotum and scutellum, smaller eyes, larger brownish yellow marking of elytra and robust protibial teeth.

##### Diagnosis.

*Bolbochromus malayensis* is similar to *Bolbochromus masumotoi*, but it can be distinguished based on the following combination of characteristics: smaller in body size (*Bolbochromus masumotoi* with larger; body length >8.0 mm); clypeal apex trapezoidal (rounded in *Bolbochromus masumotoi*); vertex with an inconspicuous carina at middle of base (a tubercle at center of frontal disc in *Bolbochromus masumotoi*); pronotal marking rounded (triangular in *Bolbochromus masumotoi*); punctures on pronotum coarse and moderately dense (fine and sparse in *Bolbochromus masumotoi*); pronotum smoothly declined anteriorly (steeply declined in *Bolbochromus masumotoi*); elytral striae coarsely punctate (finely punctate in *Bolbochromus masumotoi*); elytral intervals varying in degree of convexity (evenly convex in *Bolbochromus masumotoi*); elytral markings across interval 2–9, transversely irregular (markings across intervals 4–8, shape rounded in *Bolbochromus masumotoi*); dorsal sclerite of median lobe widened (narrow in *Bolbochromus masumotoi*).

##### Etymology.

*Bolbochromus malayensis* is the first species of the genus described from the Malay Peninsula, and the species epithet is derived from its locality.

##### Remarks.

The holotype and paratype of *Bolbochromus malayensis* were collected by a flight interception trap, which is an effective method for collecting *Bolbochromus* adults. A series of papers by Hanski and Krikken (1991), Davis (2000), Davis et al. (2001), and Li et al. (2008) demonstrated that flight interception traps are highly effective for collecting forest-dwelling bolboceratine scarabs.

## Supplementary Material

XML Treatment for Bolbochromus

XML Treatment for Bolbochromus minutus

XML Treatment for Bolbochromus nomurai

XML Treatment for Bolbochromus malayensis
